# Elevated mitochondrial activity distinguishes fibrogenic hepatic stellate cells and sensitizes for selective inhibition by mitotropic doxorubicin

**DOI:** 10.1111/jcmm.13501

**Published:** 2018-02-04

**Authors:** Priya Gajendiran, Leonel Iglesias Vega, Kie Itoh, Hiromi Sesaki, Mohammad Reza Vakili, Afsaneh Lavasanifar, Kelvin Hong, Esteban Mezey, Shanmugasundaram Ganapathy‐Kanniappan

**Affiliations:** ^1^ Division of Interventional Radiology Russell H. Morgan Department of Radiology & Radiological Science The Johns Hopkins University School of Medicine Baltimore MD USA; ^2^ Department of Cell Biology School of Medicine Johns Hopkins University School of Medicine Baltimore MD USA; ^3^ Faculty of Pharmacy and Pharmaceutical Sciences University of Alberta Edmonton Canada; ^4^ Division of Gastroenterology and Hepatology Department of Medicine The Johns Hopkins University School of Medicine Baltimore MD USA

**Keywords:** liver fibrosis, hepatic stellate cells, mitochondrial membrane potential, mitochondrial respiration, mitotropic doxorubicin

## Abstract

Activation of hepatic stellate cells (HSCs) is an integral component of the wound‐healing process in liver injury/inflammation. However, uncontrolled activation of HSCs leads to constant secretion of collagen‐rich extracellular matrix (ECM) proteins, resulting in liver fibrosis. The enhanced ECM synthesis/secretion demands an uninterrupted supply of intracellular energy; however, there is a paucity of data on the bioenergetics, particularly the mitochondrial (mito) metabolism of fibrogenic HSCs. Here, using human and rat HSCs *in vitro*, we show that the mito‐respiration, mito‐membrane potential (Δψm) and cellular ‘bioenergetic signature’ distinguish fibrogenic HSCs from normal, less‐active HSCs. *Ex vivo*, HSCs from mouse and rat models of liver fibrosis further confirmed the altered ‘bioenergetic signature’ of fibrogenic HSCs. Importantly, the distinctive elevation in mito‐Δψm sensitized fibrogenic HSCs for selective inhibition by mitotropic doxorubicin while normal, less‐active HSCs and healthy human primary hepatocytes remained minimally affected if not, unaffected. Thus, the increased mito‐Δψm may provide an opportunity to selectively target fibrogenic HSCs in liver fibrosis.

## Introduction

Liver cirrhosis represents a worldwide health problem, and epidemiology data indicate that 70–80% of cirrhotic patients will develop the primary liver cancer, hepatocellular carcinoma 1, 2. Chronic inflammation and/or injury to the liver parenchyma results in liver fibrosis that advances to cirrhosis 3. Irrespective of the causal factor, liver fibrosis/cirrhosis leads to liver failure and mortality 2, 4. Clinically, besides liver transplantation which is a limited/expensive therapy, there is no effective treatment for liver fibrosis. Consequently, several research laboratories focused on understanding the biochemical and molecular mechanisms underlying the disease progression to develop potential therapeutic strategies 5, 6, 7, 8. Despite significant progress in our understanding of liver fibrogenesis, successful clinical translation of an effective therapeutic strategy remains elusive. Thus, there is a need for the development of a viable therapeutic strategy to achieve selective and specific inhibition of fibrogenic HSCs.

Mechanistically, during liver injury, hepatic stellate cells (HSCs) get activated and undergo phenotypic alteration from a less‐active, non‐fibrogenic state to a myofibroblastic, active state 9. Activated HSCs synthesize and secrete collagen‐rich extracellular matrix (ECM) proteins which is a central event in the wound‐healing process 9, 10, 11. However, repeated or chronic cellular insults/inflammation results in uncontrolled activation of HSCs leading to continuous secretion of ECM proteins. Consequently, the excessive accumulation of ECM contributes to the transformation of normal liver parenchyma into a fibrogenic or fibrotic phenotype. As a biosynthetic process, ECM protein synthesis/secretion requires intracellular energy (*e.g*. ATP) but in abundance because of constant synthesis/secretion. Thus, the efficiency of energy producing pathways (*e.g*. glucose metabolism) is critical for the progression of fibrosis. In this context, recently, there has been a renewed interest in understanding the energy metabolism in liver fibrosis 12. Noteworthy, among the energy‐producing pathways, mitochondrial (mito) metabolism (oxidative phosphorylation [OxPhos]) and glycolysis (conversion of glucose into pyruvate followed by lactate production) have been known to be altered in early and later stages of cirrhosis 13. However, there is a lacuna in the documentation of metabolic phenotype of active, fibrogenic HSCs *per se*. Here, using human and rat HSCs *in vitro*, we show that the mito‐respiration, mito‐membrane potential (Δψm) and cellular ‘bioenergetic signature’ distinguish fibrogenic HSCs from normal, less‐active HSCs. The ‘bioenergetic signature’ of fibrogenic HSCs was also verified *ex vivo* using HSCs isolated from bile duct ligation (BDL) model of mouse and rat liver fibrosis. The striking difference in the mito‐Δψm of fibrogenic HSCs rendered them sensitive to mitotropic therapeutic, triphenylphosphonium (TPP)‐doxorubicin. Significantly, the distinctive mito‐Δψm enabled selective targeting of fibrogenic HSCs by TPP‐doxorubicin while normal HSCs and human primary hepatocytes remained minimally affected if not, unaffected. Thus, the elevated mito‐Δψm may provide a window of opportunity to selectively target fibrogenic HSCs in liver fibrosis.

## Materials and methods

### Chemicals, reagents and media

Unless otherwise mentioned, all chemicals were purchased from Sigma‐Aldrich Co. (St. Louis, MO, USA). Cell culture media, antibiotics and Geltrex were procured from Thermo Fisher Scientific Inc. (Grand Island, NY, USA). Chamber slides used for confocal microscopy were purchased from Nalgene/Nunc Inc. (Waltham, MA, USA). Collagen I, rat tail protein (Thermo Fisher Scientific), was purchased from primary antibodies such as GAPDH and F_1_–F_0_ ATPase (5E) (Santa Cruz Biotechnology Inc., CA, USA); α‐smooth muscle actin (α‐SMA) and β‐actin (Sigma‐Aldrich Co.) were purchased from respective suppliers. Secondary antibodies were purchased from Cell Signaling Technologies Inc. (Danvers, MA, USA), or Santa Cruz Biotechnology or Bio‐Rad Laboratories (Hercules, CA, USA).

### Human and rat HSCs

LX‐2, a human hepatic stellate cell line, was originally obtained from Dr. Scott Friedman (Mount Sinai School of Medicine, NY, USA), and the rat HSCs were kindly gifted by James Potter (Division of Gastroenterology and Hepatology). LX‐2 cells and rat HSCs when cultured on regular tissue culture plasticwares exhibit fibrogenic phenotype, referred as activated (fibrogenic‐) LX‐2 14. Less‐active (non‐fibrogenic) LX‐2 cells and rat HSCs were generated by culturing on the matrigel, Geltrex 14, 15, 16. The active, fibrogenic and the less‐active, non‐fibrogenic HSCs were cultured in Dulbecco's Modified Eagle's Medium (DMEM) containing 10% FBS, 0.1% antibiotic (Penicillin‐Streptomycin) and 0.1% Fungizone as described previously 17.

### Human primary hepatocytes

Human primary hepatocytes of a single donor were obtained from Lonza (Walkersville, MD, USA). All products related to the culture including growth supplements, thawing medium, maintenance medium and plating medium were also purchased from Lonza. The primary hepatocytes were revived, cultured on collagen I coated cell culture plates or dishes, and used as per supplier's instructions.

### Mouse and rat HSCs *ex vivo*

*Ex vivo* HSCs, isolated from mouse and rat models of BDL fibrosis, were generously provided by Dr. Tsukamoto (Research Center for Alcoholic Liver and Pancreatic Diseases, University of Southern California) and were prepared as described 18. *Ex vivo* HSCs were used for gene expression analysis of bioenergetic signature as described in the methods.

### Triphenylphosphonium (TPP)‐doxorubicin cytotoxicity

The synthesis of mitotropic, TPP‐conjugated doxorubicin was performed as described 19. For cytotoxicity assay, human primary hepatocytes and human HSC LX‐2 were used. The less‐active, non‐fibrogenic LX‐2 cells were generated using Geltrex as described. For the less‐active LX‐2, wells were coated with 25 μl of Geltrex 1 hr before the cell plating. In brief, cells were plated a day prior to the addition of TPP‐DOX. On the day of experiment, TPP‐DOX was added at various concentrations. Cells were treated with TPP‐DOX for 24 hrs followed by the assessment of cell viability using CyQuant NF proliferation assay kit (Thermo Fisher Scientific Inc.).

### Imaging mitochondrial function

Live imaging of mitochondria was performed with MitoTracker™ Green FM dye as described by the supplier (Thermo Fisher Scientific). Mito‐Δψm was determined using tetramethylrhodamine methyl ester (TMRM), a cationic, red‐orange fluorescent dye that is readily sequestered by active mitochondria. Hoechst dye was used to counterstain the nucleus. In brief, cells were plated in Nunc™ Lab‐Tek Chambered Coverglass and cultured in complete growth media prior to live staining. MitoTracker (200 nM) and TMRM (250 nM) staining protocols were followed as described by the supplier, and the images were captured on Zeiss LSM780 confocal microscope.

### Metabolic flux analysis

The oxygen consumption rate (OCR) and extracellular acidification rate (ECAR) of cells were measured using a Seahorse XF^96^ extracellular flux analyzer (Seahorse Bioscience, Billerica, MA, USA). In brief, LX‐2 as well as rat HSCs were plated on Seahorse XF‐96‐well plates at a density of 2 × 10^4^ per well to achieve 80–90% confluency at the time of assay. For the less‐active, non‐fibrogenic phenotype, plates were coated with Geltrex (20–25 μl volume) 1 hr before the cell plating 20 as described by the supplier (Thermo Fisher Scientific). For the experiments involving comparative analysis of human primary hepatocytes, active and less‐active LX‐2, the cells were maintained at equal densities (1–1.5 × 10^4^/well). Experimentally, cells were plated and cultured in respective growth medium in Seahorse XF‐96‐well plate. Following the overnight attachment of cells, the medium was replaced with Seahorse XF medium and protocol was followed as described by the supplier of mitostress kit (Seahorse Bioscience). Basal levels of OCR and ECAR were recorded followed by a mitochondrial stress test (1 μg/ml oligomycin, 1 μM FCCP, 1 μM rotenone/1 μM antimycin A). At the end of the assay, quantification of total cellular content was performed with CyQuant NF proliferation assay kit (Thermo Fisher Scientific Inc).

### TaqMan real‐time polymerase chain reaction (qPCR)

Gene expression analysis was performed with TaqMan Universal Master Mix II with UNG (Applied Biosystems, MA, USA) in a QuantStudio 12K Flex Real‐Time PCR System (Applied Biosystems). In brief, total RNA was extracted using Trizol reagent (Thermo Fisher Scientific) followed by RNA clean‐up using the RNeasy kit (Qiagen, Valencia, CA, USA). A known quantity of RNA (10 μg) was then subjected to reverse transcription using the high‐capacity cDNA reverse transcription kit (Applied Biosystems). The cDNAs thus synthesized were subjected to qPCR using gene‐specific TaqMan probes as listed in Table 1.

**Table 1:** List of TaqMan probes used for qPCR analysis of respective genes

Gene	Sequence/probe ID
18S	Hs03003631_g1
GAPDH	Hs99999905_m1
	Mm99999915_g1
	Rn01775763_g1
ATP5E (F_1_–F_0_ ATPase)	Mm01239887_m1
	Rn00594733_m1
ColIα1	Mm00801666_g1

### Immunoblotting

Immunoblotting was performed as described 21. In brief, cells were washed in PBS and lysed in ice‐cold RIPA lysis buffer containing protease and phosphatase inhibitor cocktails. For the lysis, cells in RIPA buffer were subjected to three cycles of freeze‐thaw followed by homogenization at 4°C using a Dounce homogenizer. The lysates were centrifuged at 12,000 × *g* for 15 min. at 4°C, the clear supernatants were collected and the protein concentrations were determined using Pierce BCA protein assay kit (Thermo Fisher Scientific). The samples were then resolved on a 4–12% Bis‐Tris gel by electrophoresis with MOPS running buffer and blotted onto PVDF membranes (Bio‐Rad, Hercules, CA, USA) followed by immunoblotting with specific antibodies. Immune complexes were visualized by ECL‐detection kit (GE Healthcare).

### Glucose uptake assay

Glucose uptake was determined as described 22 with relevant modifications. LX‐2 cells growing in log phase were seeded in 6‐well plates a day before the glucose assay (for less‐active phenotype, the Geltrex coating was performed as described above). On the day of glucose uptake assay, cells were washed twice and incubated with Opti‐MEM media supplemented with 0.2% BSA for 2 hr. Then, the Opti‐MEM medium was replaced with PBS containing 0.1 mM 2‐deoxyglucose and 1 μCi ^3^H‐2‐deoxyglucose (PerkinElmer, Boston, MA, USA) in the presence or absence of 25 μM cytochalasin B to determine non‐specific glucose uptake. Note, the cytochalasin B was added to cells 15 min. prior to the addition of 2‐deoxyglucose/^3^H‐2‐deoxyglucose. The glucose uptake assay was terminated 15 min. after the incubation with 2‐deoxyglucose/^3^H‐2‐deoxyglucose. Next, cells were washed with ice‐cold PBS buffer twice followed by complete lysis and solublization in 0.1% SDS. Intracellular glucose was quantified by measuring ^3^H‐radioactivity in the scintillation fluid using a *β*‐scintillation counter. The non‐specific glucose uptake (in the presence of cytochalasin B) was determined and deducted to arrive at specific glucose uptake; data were normalized for protein concentration which was determined using the Pierce BCA protein assay (Thermo Fisher scientific Co.). All procedures involving ^3^H‐2‐deoxyglucose samples were used only by authorized personnel and appropriate radioactive decontaminations; containments were followed strictly according to the Johns Hopkins Radiation Safety rules and regulations.

### Statistical analysis

All experimental data represent mean and standard error of at least triplicate samples. Data were analysed by Student's *t*‐test.

## Results and discussion

**Figure 1:**
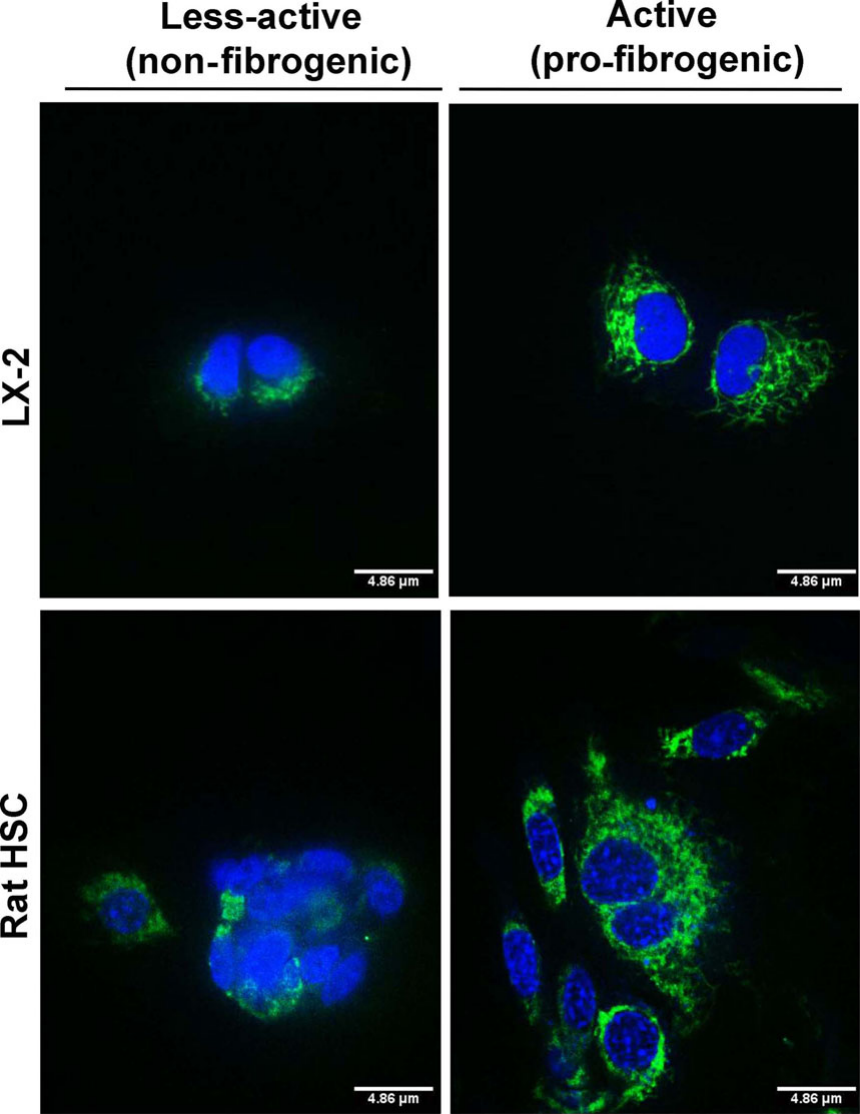
Mitochondrial capacity of the human HSC, LX‐2 and rat HSCs under less‐active (non‐fibrogenic) and active (pro‐fibrogenic) conditions. HSCs of non‐fibrogenic and pro‐fibrogenic phenotypes were stained with the MitoTracker fluorescent probe (an indicator of mitochondrial function). The green fluorescence indicates functionally active mitochondria which is abundant and highly prominent in active, fibrogenic HSCs. Although the less‐active cells show some green fluorescence, it is evident that the fluorescence is diffused and merged onto the nuclear stain (blue colour by Hoechst stain) resulting in a cloudy bluish‐green signal which indicates a functionally less‐active mitochondria. Representative images from triplicate experiments are shown.

**Figure 2:**
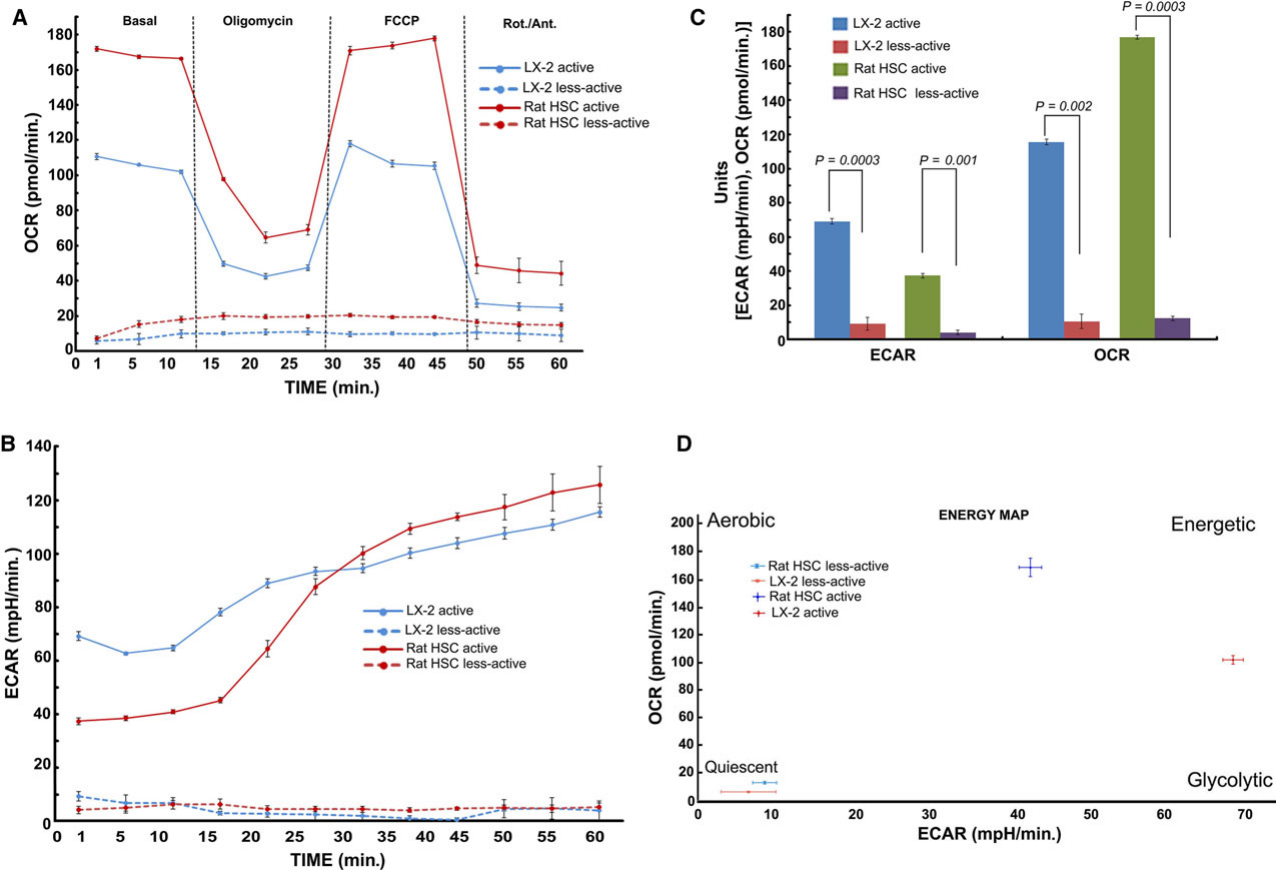
Metabolic flux analysis showing the elevated mitochondrial activity in active (fibrogenic) HSCs of human (LX‐2) and rat origin. **A**, Time course of oxygen consumption rate (OCR), **B**, the extracellular acidification rate (ECAR), **C**, quantitative analysis and **D**, the energy map based on the ratio of OCR and ECAR as determined using Seahorse XF metabolic flux analyzer. Overall, the basal respiration, oligomycin insensitive respiration (also known as leaking), FCCP‐induced respiration and non‐mitochondrial (Rotenone/Antimycin A) respiration were shown. The total cellular content was quantified using CyQuant NF proliferation assay kit as mentioned in the Methods section. Data represent Mean ± S.D., *n* = 5 wells per group.

**Figure 3:**
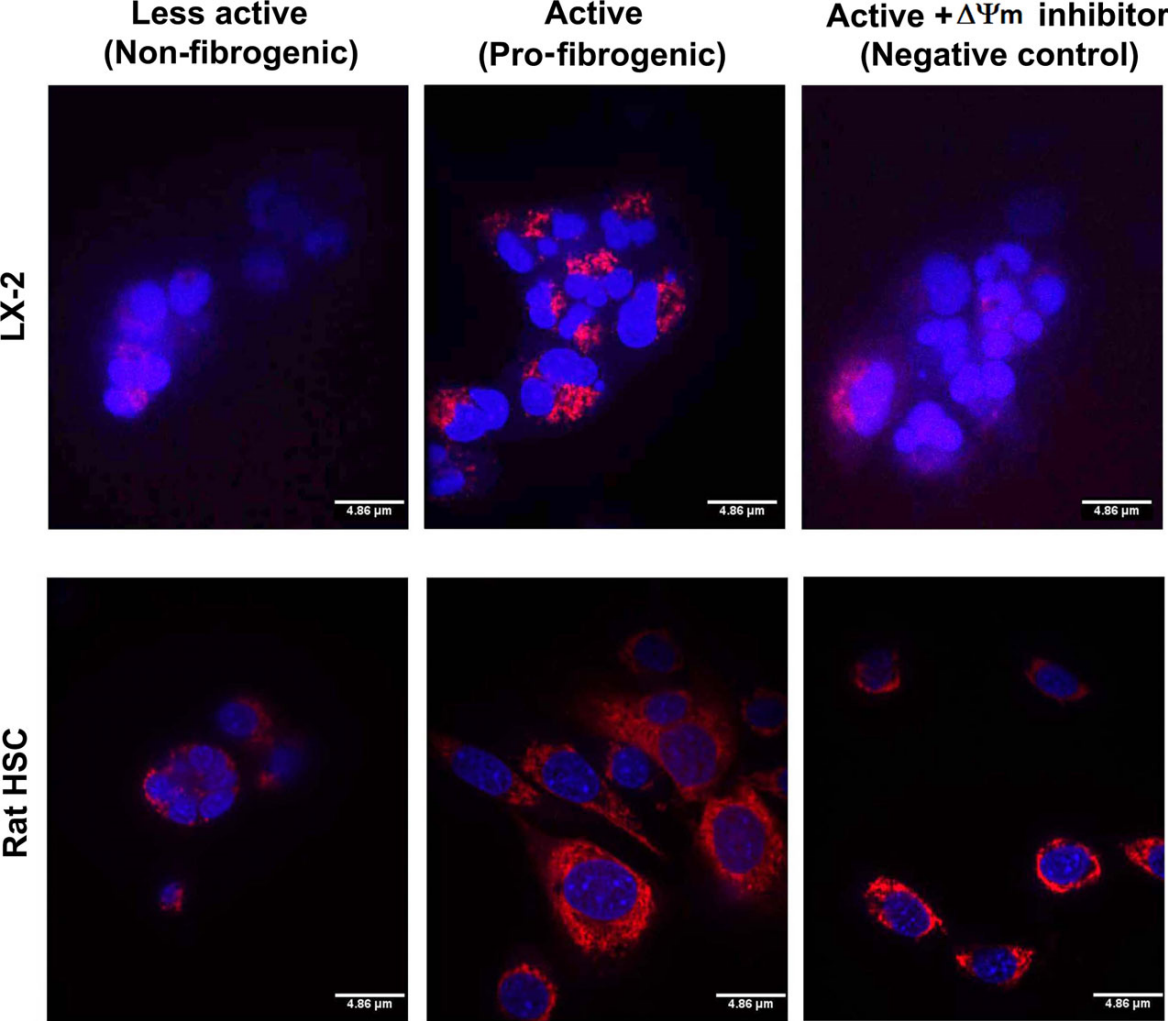
Mitochondrial membrane potential is distinctively amplified in fibrogenic HSCs. LX‐2 and rat HSCs of less‐active, non‐fibrogenic phenotype as well as active, fibrogenic phenotype were stained using the TMRM fluorescent probe (a mitochondrial membrane potential indicator). The red fluorescence indicates the mitochondrial membrane potential (ΔΨm) which is pronounced in active, fibrogenic LX‐2 and rat HSCs. A negative control (treated with FCCP inhibitor) that decreased mito‐ΔΨm in active, fibrogenic LX‐2 and rat HSCs is shown to demonstrate the specificity and distinctiveness of ΔΨm staining. Although the less‐active cells and negative control show some red fluorescence, it is evident that the fluorescence is diffused and merged onto the nuclear stain (blue colour by Hoechst stain) resulting in a cloudy purple signal, which indicates a reduced mito‐ΔΨm. Representative images from duplicate experiments are shown. Scale = 4.86 μm.

Alteration in the metabolic phenotype, particularly, the mitochondrial metabolism has recently been reported in cirrhosis, yet data on fibrogenic HSCs, the critical determinant of fibrosis, remain unknown 12, 13. Our data show an increase in the mitochondrial activity of pro‐fibrogenic HSCs of human (LX‐2) and rat origin (Fig. 1). The enhanced mitochondrial function was further substantiated by the metabolic flux analysis. Results from the analysis of mitochondrial respiration showed a striking elevation in the oxygen consumption rate (OCR) of fibrogenic HSCs compared to the normal, less‐active counterpart as demonstrated from human HSC, LX‐2 and rat HSCs (Fig. 2A and C). The significant increase in mito‐respiration also reflected in the extracellular acidification rate (ECAR) (Fig. 2B and C). Energy map based on the OCR and ECAR demonstrated that fibrogenic HSCs have higher energy level (bioenergetic capacity) perhaps to corroborate the functional demand (Fig. 2D). With the finding that the mitochondrial activity is enhanced in fibrogenic HSCs, we next investigated the mito‐Δψm. As shown in Figure 3, the fibrogenic HSCs have markedly increased mito‐Δψm compared to the less‐active HSCs of both human and rat origin.

**Figure 4:**
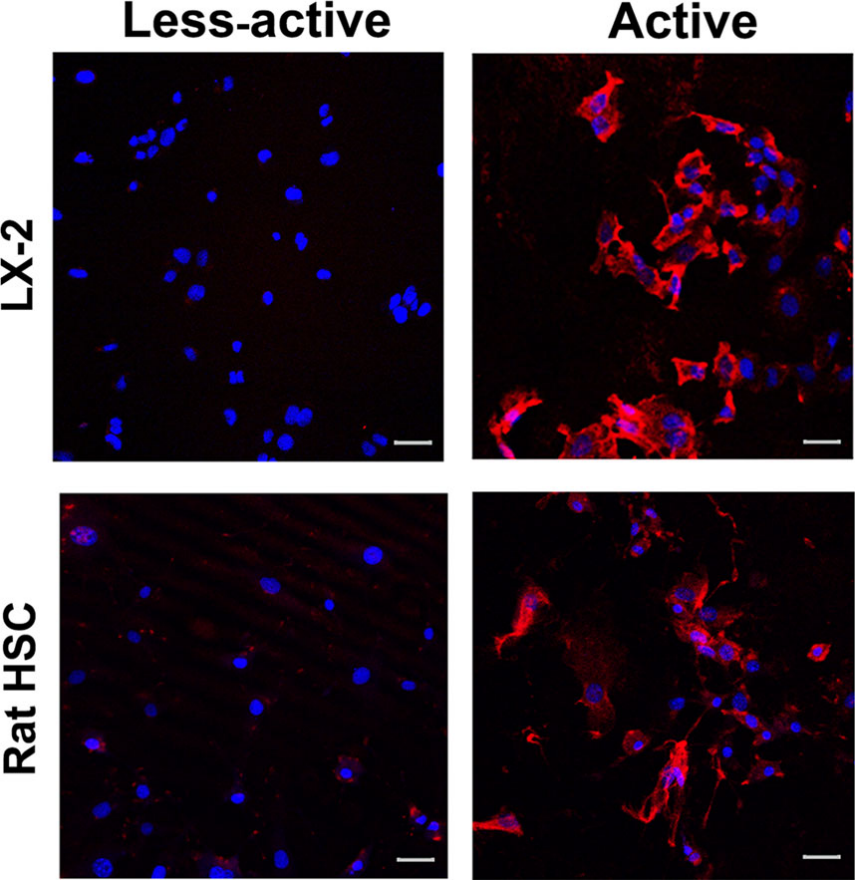
Expression of α‐SMA in LX‐2 and rat HSCs. Immunofluorescent images showing the level of expression of fibrogenic marker, α‐SMA in fibrogenic LX‐2 and rat HSCs. Scale = 20 μm. The LX‐2 images were reproduced from Karthikeyan *et al*. 20 with the permission of Elsevier.©

**Figure 5:**
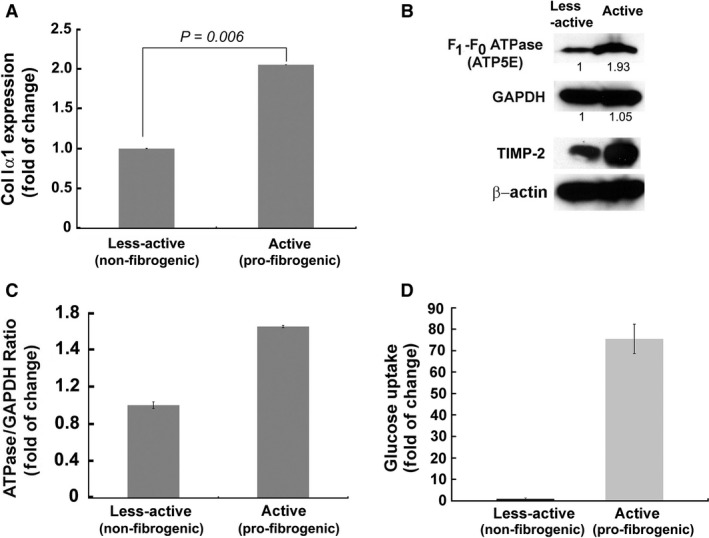
Fibrogenic, active LX‐2 demonstrate higher bioenergetic ratio than the less‐active LX‐2. **A**, Verification of the fibrogenic phenotype of active LX‐2 cells by TaqMan qPCR quantification of *ColIα1*, one of the biochemical markers of fibrogenesis. **B**, Immunoblot showing increased expression of F_1_–F_0_ ATPase in active (fibrogenic) LX‐2. TIMP‐2 confirmed the pro‐fibrogenic phenotype of active LX‐2 cells. β‐actin is shown as the loading control. **C**, The bar graph represents densitometry data. **D**, Bar graph showing the rate of ^3^H‐2‐deoxyglucose uptake in less‐active and active LX‐2 cells.

Next, we verified the ‘bioenergetic signature’ of fibrogenic phenotype. Bioenergetic signature is the ratio between the rate of glycolysis (as measured by the expression of the glycolytic enzyme, glyceraldehyde‐3‐phosphate dehydrogenase, GAPDH) and OxPhos (as indicated by the level of expression of mitochondrial F_1_–F_0_ ATPase) 23. Prior to the determination of the bioenergetic index, the fibrogenic activation or the less‐active phenotype of LX‐2 and rat HSCs were also verified by immunostaining for α‐smooth muscle actin (SMA), one of the markers of fibrogenic phenotype. Figure 4 shows that α‐SMA expression has elevated in active HSCs of rat and human (LX‐2) origin. Next, the immunoblot data revealed a marked increase in the expression of F_1_–F_0_ ATPase (Fig. 5), and the ratio between F_1_–F_0_ ATPase and GAPDH demonstrated a significant elevation in the bioenergetic signature (Fig. 5). The fibrogenic phenotype of activated HSCs were confirmed by the marker collagenIα1 (ColIα1) (Fig. 5A) as well as the expression of TIMP‐2 (Fig. 5B). Earlier, we have demonstrated the validation of fibrogenic phenotype of activated HSCs (LX‐2) using additional markers (*e.g*. α‐smooth muscle actin, cytokeratin‐18) 20. Densitometry data show an increase in the ratio between ATPase and GAPDH expression in pro‐fibrogenic (active) LX‐2 cells compared to the less‐active LX‐2 (Fig. 5C). As the increase in mitochondrial activity and GAPDH expression are likely to augment the rate of glucose hydrolysis, we then investigated the rate of glucose uptake. Determination of rate of glucose uptake using ^3^H‐2‐deoxyglucose revealed a marked increase in glucose utilization indicating overall elevation in the rate of glucose metabolism (Fig. 5D).

**Figure 6:**
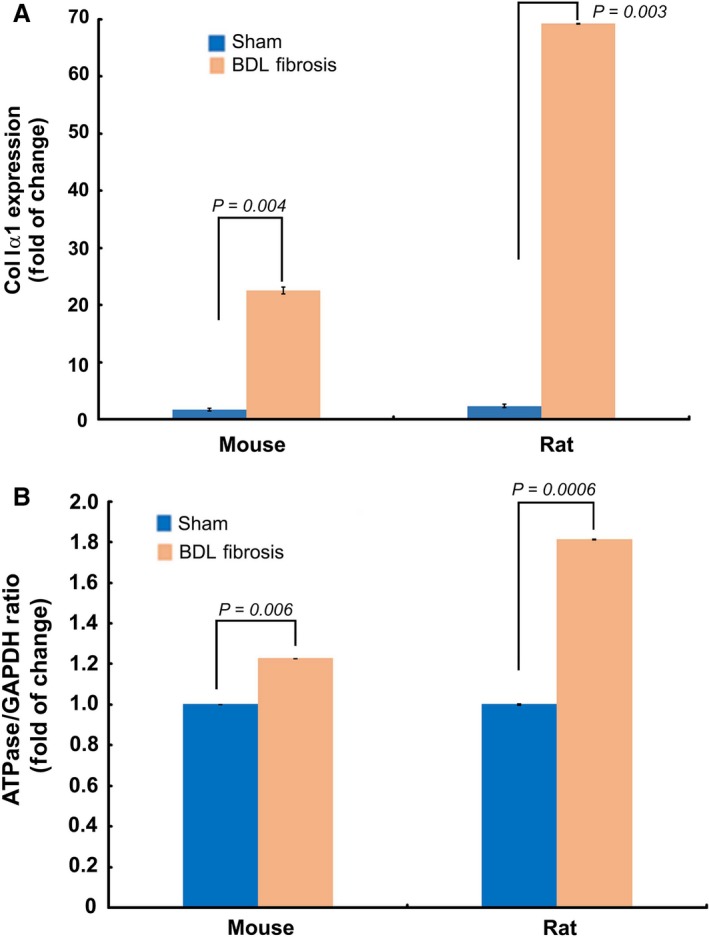
Fibrogenic, active HSCs demonstrate higher bioenergetic ratio than the less‐active HSCs *ex vivo*. **A**, TaqMan qPCR quantification of *ColIα1* expression verified the fibrogenic phenotype of HSCs *ex vivo*, from the mouse and rat models of BDL fibrosis. **B**, TaqMan qPCR data showing increased bioenergetic ratio of fibrogenic HSCs *ex vivo*, from the mouse and rat models of BDL fibrosis. Data represent Mean ± S.E. (*n* = 3).

We next asked whether fibrogenesis‐related increase in the mito‐activity affects the ‘bioenergetic signature’ *in vivo*. We used normal and fibrogenic HSCs isolated (*ex vivo*) from the mouse and rat models of BDL liver fibrosis to determine fibrogenesis‐related alteration in the bioenergetic signature. These *ex vivo* HSCs were generously provided by Dr. Tsukamoto (Research Center for Alcoholic Liver and Pancreatic Diseases, University of Southern California). TaqMan qPCR analysis of the expression of GAPDH and F_1_–F_0_ ATPase further confirmed the fibrogenesis‐related increase in bioenergetic signature of HSCs (Fig. 6). ColIα1 expression (Fig. 6) was quantified to confirm the fibrogenic phenotype of these *ex vivo* HSCs.

**Figure 7:**
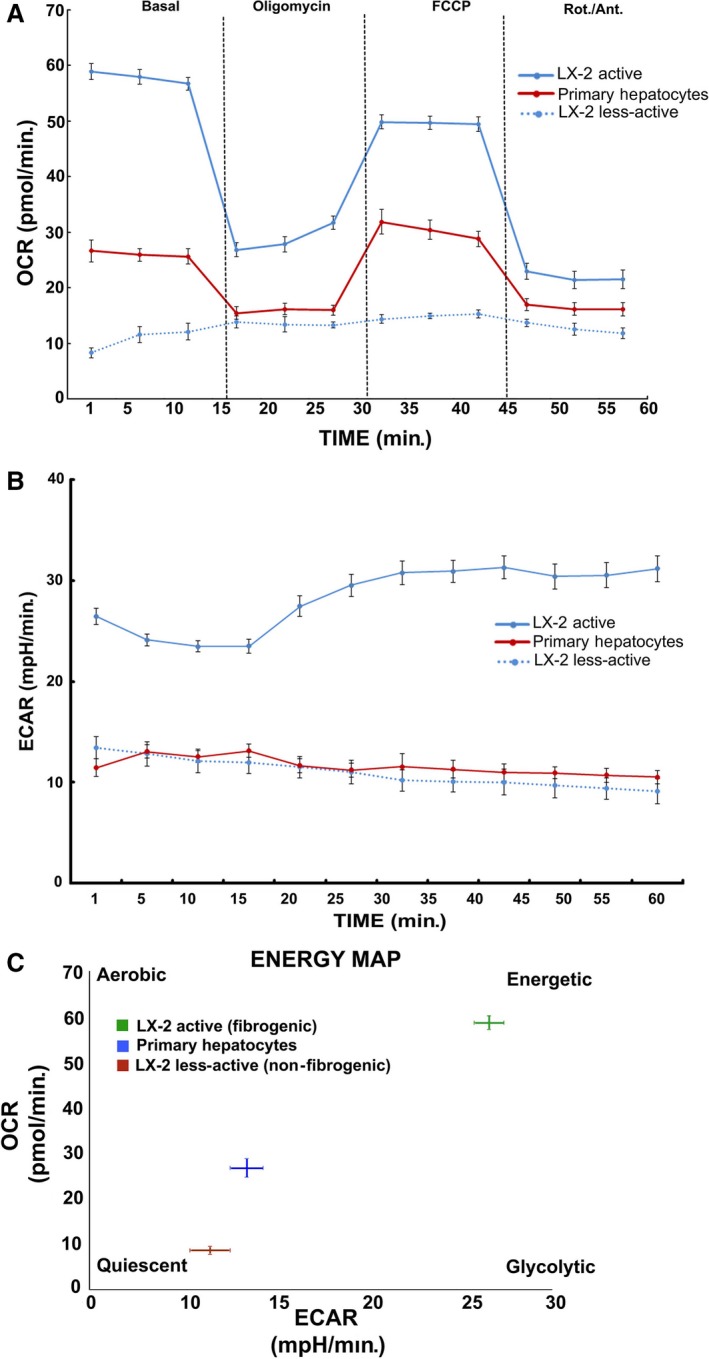
Metabolic flux analysis show increased mitochondrial activity of fibrogenic LX‐2 compared to human primary hepatocytes. **A**, Time course of oxygen consumption rate (OCR), **B**, the extracellular acidification rate (ECAR) and **C**, the energy map based on the ratio of OCR and ECAR as determined using XF‐96 flux analyzer. Overall, the basal respiration, oligomycin insensitive respiration (also known as leaking), FCCP‐induced respiration and non‐mitochondrial (Rotenone/Actinomycin D) respiration were shown. Mean ± S.D., *n* = 5 wells per group.

**Figure 8:**
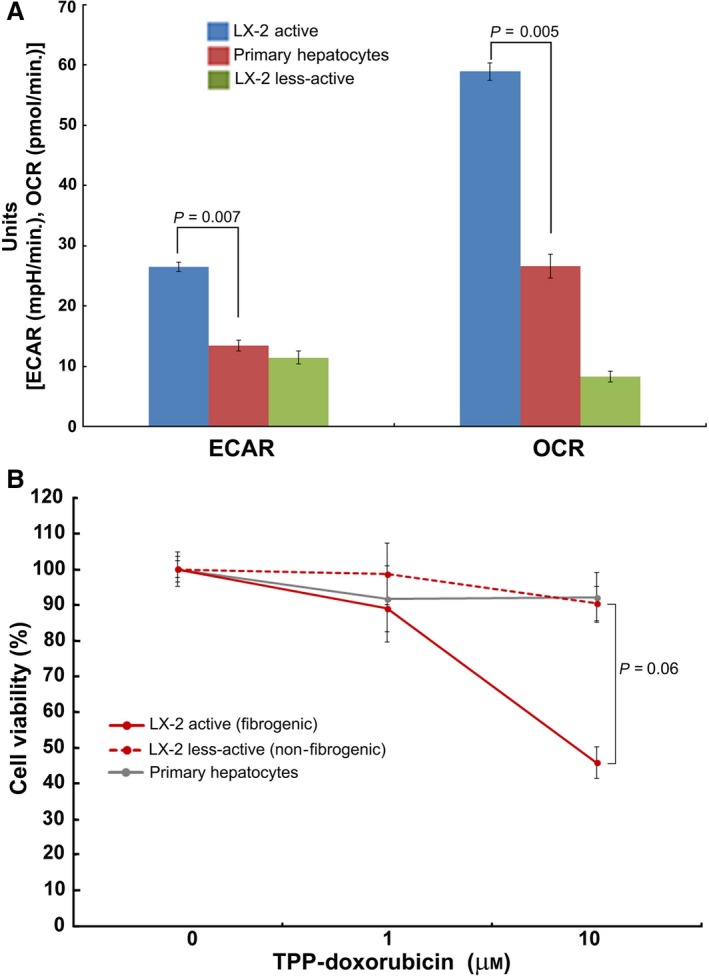
Active LX‐2 mitochondrial activity markedly elevated than human primary hepatocytes and sensitive to TPP‐doxorubicin. **A**, Comparative analysis of the extracellular acidification rate (ECAR) and oxygen consumption rate (OCR) of active (fibrogenic) LX‐2 cells showing higher mitochondrial activity than the less‐active LX‐2 and human primary hepatocytes. Mean ± S.E., *n* = 5. **B**, The active, fibrogenic LX‐2 cells are sensitive to TPP‐doxorubicin treatment compared to the less‐active LX‐2 and human primary hepatocytes.

Based on the data that fibrogenic phenotype correlates with elevated mitochondrial activity and higher mito‐Δψm, we explored whether the distinctively elevated, functional activation of mitochondria could be exploited for selective targeting of fibrogenic HSCs. Precisely, we examined the relevance of mitotropic agents that rely on mito‐Δψm to specifically target mitochondria. For example, TPP has been known to selectively target cells with increased mito‐Δψm 19, 24, 25. In other disease conditions like cancer such TPP‐conjugated therapeutics have been found to promote desired therapeutic effects (*e.g*. TPP‐doxorubicin 19, TPP‐dichloroacetate 24). Hence, we hypothesized that the marked increase in mito‐Δψm of fibrogenic HSCs may provide an opportunity for selective targeting. Although the primary objective is to test the efficacy of therapeutic targeting of fibrogenic HSCs, it is also critical to verify the feasibility of selective inhibition to eliminate potential concerns related to undesirable toxicity. Particularly, the preservation of healthy cells (*e.g*. primary hepatocytes) of the liver is of paramount importance while treating cirrhotic or fibrotic liver. Thus, while the fibrogenic HSCs showed elevated mitochondrial activity compared to normal, less‐active HSCs, it is imperative to determine the level of mitochondrial activity in normal liver cells (*e.g*. primary hepatocytes). Accordingly, we evaluated the mitochondrial function of human hepatocytes. Comparative analysis of the metabolic flux of human hepatocytes with fibrogenic and non‐fibrogenic LX‐2 revealed that the fibrogenic LX‐2 cells have markedly higher mitochondrial activity as evident by OCR, ECAR and the energy map (Figs 7 and 8A). With the evidence that the mitochondrial activity of fibrogenic HSCs (LX‐2 is significantly elevated than healthy human hepatocytes, we next evaluated the cytotoxicity TPP‐doxorubicin *in vitro*. As mentioned earlier, use of TPP‐doxorubicin is a therapeutically relevant approach that relies on mito‐Δψm for its selective, intracellular targeting of mitochondria. Notably, fibrogenic HSCs were markedly sensitive to TPP‐doxorubicin, while the less‐active HSCs and primary hepatocytes remained minimally affected if not, unaffected (Fig. 8B). However, the data represent *in vitro* findings, and it is critical to evaluate the findings *in vivo* for further progress. Nonetheless, our report provides first evidence that fibrogenic HSCs have distinctive mito‐Δψm that may be exploited for selective inhibition.

Earlier, we demonstrated that deregulation of energy metabolism affects F_1_–F_0_ ATPase to promote anti‐fibrotic effects 20. Here, we demonstrate that mito‐Δψm distinguishes fibrogenic HSCs from normal HSCs and healthy primary hepatocytes, thus providing us an opportunity for selective targeting. Mito‐Δψm is the biochemical signature of mitochondrial activity that is critical for ATP synthesis. Thus, the elevated mito‐Δψm indicates higher mitochondrial activity which corroborates the necessity to meet the demands (*e.g*. ECM synthesis) of fibrogenic HSCs. Targeting mitochondria has been contemplated as a potential therapeutic strategy for cirrhosis 26, 27, 28, 29, 30. However, its clinical relevance remains a challenge because of inevitable systemic toxicity. The ubiquitous nature of mitochondria necessitates any viable or potential anti‐mitochondrial approach to be selective in targeting cirrhotic or fibrotic liver. Our results provide evidence for the prominence of mitochondrial activity and a marked elevation in mito‐Δψm of fibrogenic HSCs that will enable us to develop strategies to selectively inhibit fibrogenic HSCs. Our findings may stimulate and rationalize further studies to delineate the role of mito‐Δψm between early or later stages of fibrosis and to verify whether a therapeutic opportunity exists to treat advanced liver cirrhosis as well.

As the normal, less‐active (non‐fibrogenic) HSCs undergo functional and phenotypic expansion there is a demand for abundant and continuous supply of intracellular energy. During such energy demand, eukaryotic cells adopt altered metabolic pathways referred as ‘metabolic reprogramming’. For example, normal T cells upon activation undergo a metabolic switch to ‘aerobic glycolysis’—the process of conversion of glucose into pyruvate followed by lactate production. Similarly, normal brain has been reported to adopt the aerobic glycolysis which mitigates the generation of intracellular ROS 31. Thus, although less‐efficient in terms of the production of total number of ATP molecules, glycolysis has a higher rate of glucose oxidation that compensates for reduced energy output. More importantly, it reduces the mitochondrial burden. Similarly, it has been established that highly proliferative cells such as cancer cells also adopt the ‘glycolytic’ phenotype as shown by elegant reviews 32, 33, 34, 35. Thus, irrespective of the cell type or the disease condition, an increase in energy demand in general facilitates metabolic shift to glycolysis, a rapid energy‐producing pathway. Interestingly, our data show that despite a highly proliferative and biosynthetically active (*e.g*. ECM synthesis) phenotype, fibrogenic HSCs exhibit active mitochondrial metabolism rendering them sensitive to targeting by mitotropic anti‐mitotic agent (e.g. TPP‐doxorubicin). Future studies might shed light on the role of mitochondria, besides energy metabolism, in the regulation of fibrogenesis.

## Conflict of interest

The authors confirm that there are no conflicts of interest.
